# Modeling Cellular Noise Underlying Heterogeneous Cell Responses in the Epidermal Growth Factor Signaling Pathway

**DOI:** 10.1371/journal.pcbi.1005222

**Published:** 2016-11-30

**Authors:** Kazunari Iwamoto, Yuki Shindo, Koichi Takahashi

**Affiliations:** 1 Laboratory for Biochemical Simulation, RIKEN Quantitative Biology Center, Suita, Osaka, Japan; 2 Institute for Advanced Biosciences, Keio University, Tsuruoka, Yamagata, Japan; University of Virginia, UNITED STATES

## Abstract

Cellular heterogeneity, which plays an essential role in biological phenomena, such as drug resistance and migration, is considered to arise from intrinsic (i.e., reaction kinetics) and extrinsic (i.e., protein variability) noise in the cell. However, the mechanistic effects of these types of noise to determine the heterogeneity of signal responses have not been elucidated. Here, we report that the output of epidermal growth factor (EGF) signaling activity is modulated by cellular noise, particularly by extrinsic noise of particular signaling components in the pathway. We developed a mathematical model of the EGF signaling pathway incorporating regulation between extracellular signal-regulated kinase (ERK) and nuclear pore complex (NPC), which is necessary for switch-like activation of the nuclear ERK response. As the threshold of switch-like behavior is more sensitive to perturbations than the graded response, the effect of biological noise is potentially critical for cell fate decision. Our simulation analysis indicated that extrinsic noise, but not intrinsic noise, contributes to cell-to-cell heterogeneity of nuclear ERK. In addition, we accurately estimated variations in abundance of the signal proteins between individual cells by direct comparison of experimental data with simulation results using Apparent Measurement Error (AME). AME was constant regardless of whether the protein levels varied in a correlated manner, while covariation among proteins influenced cell-to-cell heterogeneity of nuclear ERK, suppressing the variation. Simulations using the estimated protein abundances showed that each protein species has different effects on cell-to-cell variation in the nuclear ERK response. In particular, variability of EGF receptor, Ras, Raf, and MEK strongly influenced cellular heterogeneity, while others did not. Overall, our results indicated that cellular heterogeneity in response to EGF is strongly driven by extrinsic noise, and that such heterogeneity results from variability of particular protein species that function as sensitive nodes, which may contribute to the pathogenesis of human diseases.

## Author Summary

Individual cell behaviors are controlled by a variety of noise, such as fluctuations in biochemical reactions, protein variability, molecular diffusion, transcriptional noise, cell-to-cell contact, temperature, and pH. Such cellular noise often interferes with signal responses from external stimuli, and such heterogeneity functions in induction of drug resistance, survival, and migration of cells. Thus, heterogeneous cellular responses have positive and negative roles. However, the regulatory mechanisms that produce cellular heterogeneity are unclear. By mathematical modeling and simulations, we investigated how heterogeneous signaling responses are evoked in the EGF signaling pathway and influence the switch-like activation of nuclear ERK. This study demonstrated that cellular heterogeneity of the EGF signaling response is evoked by cell-to-cell variation of particular signaling proteins, such as EGFR, Ras, Raf, and MEK, which act as sensitive nodes in the pathway. These results suggest that signaling responses in individual cells can be predicted from the levels of proteins of sensitive nodes. This study also suggested that proteins of sensitive nodes may serve as cell survival mechanisms.

## Introduction

Intracellular signaling pathways must respond appropriately to various signals from the external environment. However, a variety of noise inside and outside of the cells can evoke heterogeneous responses in individual cells even when exposed to the same stimuli [1,2]. Although such heterogeneity interferes with a precise signaling response, it often plays essential roles in biological functions. Examples include diverse responses between amoeba cells that can undergo collective chemotaxis, and enhancement of signal entrainment in NF-κB response over a wider range of dynamic inputs by cellular noise [3,4]. Cellular noise is categorized into intrinsic and extrinsic noise [5–7]. Intrinsic noise is generally evoked by small numbers of molecules, representing fluctuations in biochemical reactions, transcriptional noise, molecular diffusion, etc. One of the best-known examples is the stochastic gene expression in *Escherichia coli* [5]. On the other hand, extrinsic noise is defined by the differences in amounts of proteins in individual cells (protein variability) and external physical environments, such as cell-to-cell contact, cell cycle phase and cell shape. The relationships between heterogeneous cellular responses and extrinsic noise in various signaling pathways have been reported [4,8–10]. However, the mechanistic effects of these types of noise to determine heterogeneity of signal responses are unclear. In this study, using mathematical modeling and simulations, we determined how cellular noise regulates heterogeneous cell responses, focusing on the epidermal growth factor (EGF) signaling pathway as an example.

The EGF signaling pathway regulates cell growth, proliferation, differentiation, and apoptosis [11,12]. EGF ligands bind to EGF receptors (EGFR), and the signal is transmitted to an intracellular biochemical reaction network. This signal transduction eventually phosphorylates extracellular signal-regulated kinase (ERK) and causes transient accumulation of ERK at the nucleus [13,14]. The EGF dose response of phosphorylated ERK shows a graded response [10,15–17]. However, it was recently reported that the dose response of nuclear ERK activity is in fact switch-like [14]; a threshold mechanism regulated by ERK may be involved in cell fate decision. The switch-like behavior is more sensitive to perturbations than the graded response [18], and hence the effect of biological noise is considered to be critical to determine nuclear ERK activity. In fact, heterogeneous cell responses in nuclear ERK have been observed [13,14]. To determine how such heterogeneity in nuclear ERK response is evoked, we performed mathematical modeling and simulation analysis of the EGF signaling pathway. We developed a mathematical model of the EGF signaling pathway integrating feedback regulation between ERK and nuclear pore complex (NPC), which is essential for switch-like activation of nuclear ERK, and previously developed mathematical schemes [19–21]. We also developed a new method to compare simulation results with experimental data by estimating Apparent Measurement Error (AME). Finally, we elucidated how intrinsic and extrinsic noise regulate heterogeneity in nuclear ERK responses.

## Model

### Overview of the model

**Fig 1:**
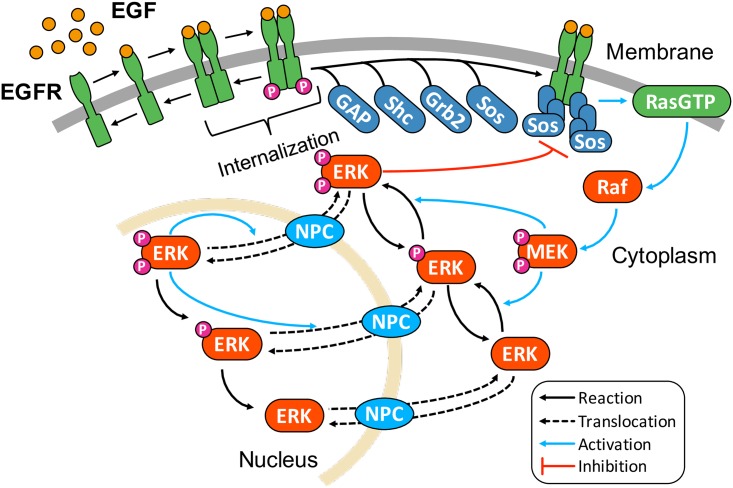
Simplified reaction network of EGF signaling pathway model. Details of reactions are shown in S1 Fig.

As shown in Fig 1, we developed a novel mathematical model based on the biological reaction networks of the EGF signaling pathway integrated with autoregulatory control of ERK translocation. The details of our model are as follows.

### EGF signaling pathway

The reaction scheme of EGF signaling pathway is based on several published mathematical models [19–21]. EGF signaling is initiated by binding between EGF ligands and EGFR on the cell membrane, and EGF–EGFR complexes are subsequently dimerized and autophosphorylated [22–24]. Phosphorylated EGFR dimer (pEGFR) transmits the signal through two pathways, i.e., the src homology and collagen protein (Shc)-independent/dependent pathways. Shc bound to pEGFR associates with growth factor receptor-bound protein 2 (Grb2), while Grb2 can directly associate with pEGFR [25,26]. Grb2 in both pathways recruits Son of Sevenless (Sos) from the cytoplasm to the membrane, which binds to the membrane-anchored protein Ras [27,28]. This association leads to exchange of guanosine diphosphate of Ras (RasGDP) for guanosine triphosphate (RasGTP). The inactivation of RasGTP is mediated by GTPase activating protein (GAP) [19,20,29]. The details of this reaction scheme are shown in S1A Fig. Although little is known about the detailed reaction processes involved in Raf activation, a model of Raf activation was recently proposed based on single-molecule observations [30,31]. In this model, both RasGDP and RasGTP can associate with Raf. However, the association rate between Raf and RasGTP was higher than that of RasGDP. Only the RasGTP–Raf complex is able to activate Raf through an intermediate state. Kinetic parameters in the reactions were estimated from experimental data [31]. This activation scheme of Raf is included in our model (S1B Fig). Activated Raf doubly phosphorylates cytoplasmic MEK (ppMEK), and subsequently ppMEK also doubly phosphorylates ERK (ppERK). In addition, ppERK inhibits Sos through phosphorylation, which acts as negative feedback in EGF signaling [32,33]. We assumed that Raf, MEK, and ERK are dephosphorylated by different phosphatases [20]. All biochemical reactions related to the EGF signaling pathway in our model are shown in S1A and S1B Fig.

### Autoregulatory control of nuclear translocation of ERK

ERK transiently translocates into the nucleus through binding with NPC after EGF stimulation [13]. Several regulatory mechanisms of ERK translocation have also been proposed [14,34,35]. ERK-mediated phosphorylation of NPC reduces the nuclear accumulation of importin-beta that transports several proteins, including ERK, from the cytoplasm to the nucleus [34,35], suggesting that activated ERK may potentially regulate its own translocation. In addition, we recently demonstrated that ERK-mediated phosphorylation of NPC is involved in the switch-like behavior of nuclear ERK translocation [14]. Based on these biological findings, we developed a new mathematical model to describe ERK translocation (S1C Fig). In our model, cytoplasmic and nuclear ERK bind to NPC and translocate between the cytoplasm and the nucleus. An NPC has multiple phosphorylation sites for ERK in FG nucleoporins, which regulate the permeability barrier properties of the NPC [34,36]. The dynamic behaviors of such multiple phosphorylation systems have been reproduced using two-step reaction models. For example, retinoblastoma tumor suppressor protein regulated by multiple phosphorylation was modeled using two-step phosphorylation, i.e., considering non-, hypo-, and hyperphosphorylated forms in several models [37–39]. Therefore, we introduced two phosphorylation states of NPC that are mediated by nuclear ppERK into our model (pNPC and ppNPC in S1C Fig). Further, it was reported that the translocation rate of ERK was dependent on phosphorylation states of both ERK and NPC [14,34,40]. To clarify the effect of NPC phosphorylation on the ERK translocation, we assumed the following: the translocation rates of non-phosphorylated and phosphorylated ERK are different among the phosphorylation states of NPC, both NPC and pNPC mediate the translocation of phosphorylated ERK from cytoplasm to nucleus, and ppNPC allows unidirectional translocation of ERK, from nucleus to cytoplasm (S4 Table). Overall, in our model, cytoplasmic ERK is phosphorylated and translocates into the nucleus transiently after EGF stimulation. Thereafter, nuclear ppERK phosphorylates NPC in a two-step process, which finally induces the export of phosphorylated ERK from the nucleus to the cytoplasm (Fig 1).

### Mathematical model

Our model consists of 78 chemical species and 150 biochemical reactions. The initial conditions, i.e., the number of molecules, of each species are shown in S1 Table. ERK and its phosphatases were considered to be distributed in both the cytoplasm and the nucleus (Fig 1). The biochemical reaction processes, association, dissociation, phosphorylation, dephosphorylation, and degradation, were described by mass-action law (S2 Table). Details of the simulation method and how to determine the kinetic parameters are described in the Materials and Methods.

## Results

### Different responses of phosphorylated and nuclear ERK

**Fig 2:**
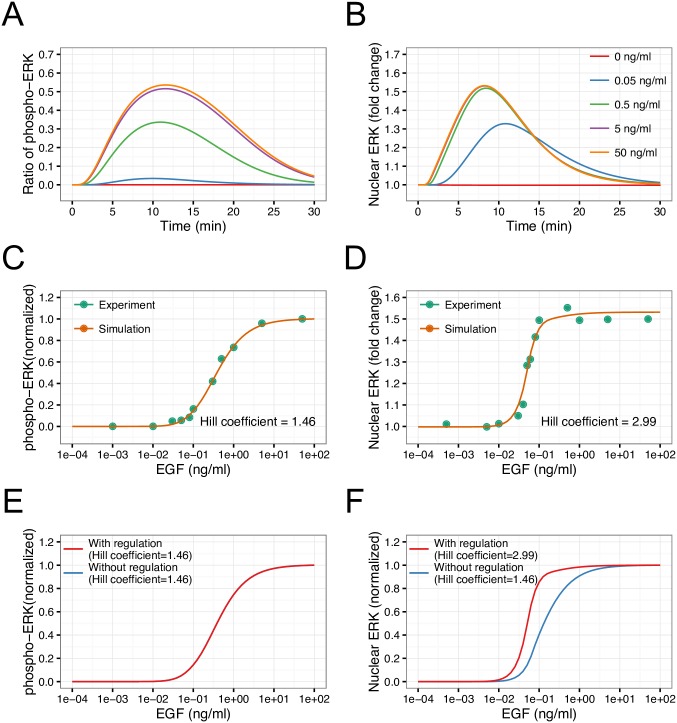
Validation of EGF signaling pathway model. The time courses of changes in (A) phosphorylated and (B) nuclear ERK levels at different concentrations of EGF (0–50 ng/mL) are shown. EGF dose response of peak levels at (C) phosphorylated and (D) nuclear ERK were calculated from deterministic simulations. Points and lines represent observed data [14] and simulation results, respectively. The effects of ERK-mediated regulation of NPC on the dose response of (E) phosphorylated and (F) nuclear ERK are shown. The Hill coefficients were obtained by curve fitting of simulation results.

To confirm the biological validity of our mathematical model, we first implemented deterministic simulations. Here, pERK represents the total amount of singly/doubly phosphorylated ERK, including their complexes, and nERK represents the fold change in nuclear ERK, defined as the ratio of the total amount of nuclear ERK to the initial value. Simulated time courses of both pERK and nERK showed transient dynamics, and peak levels increased with elevated EGF concentration (Fig 2A and 2B). These dynamics were consistent with the typical dynamics after EGF stimulation observed in cell lines of various types [13,14,19,40]. Next, we calculated the EGF dose response of peak levels of pERK and nERK (Fig 2C and 2D), which showed good agreement with the experimental data [14]. The dose response of pERK showed a graded pattern (Hill coefficient = 1.46), while that of nERK showed switch-like behavior (Hill coefficient = 2.99). To confirm that this difference was caused by ERK-mediated regulation of NPC, we performed simulation without the regulation from ERK to NPC. To remove this regulation, the kinetic parameters related to ERK-mediated phosphorylation of NPC were set to zero (reaction number 137–144 in S1C Fig, bottom right). As shown in Fig 2E and 2F, the dose response of nERK changed from switch-like to graded, and the Hill coefficient of nERK corresponded to that of pERK (Hill coefficient = 1.46). This result indicated that ERK-mediated phosphorylation of NPC is responsible for the switch-like response of nERK. The ERK-mediated phosphorylation of NPC accelerates a nuclear export of ERK in our model, establishing a negative autoregulation of nuclear ERK (Fig 1). To investigate the mechanism by which the negative autoregulation changed dynamics of nERK, the dose response of nERK at fold change level was shown in S2 Fig. The negative autoregulation of nuclear ERK drastically reduced the maximum fold change level of nuclear ERK, resulting in decreasing the range of effective concentration (EC) 10 and EC90 (S2 Fig). While the level of EC90 was decreased from 1.00 to 0.12 by the negative autoregulation, the level of EC10 did not change. This is because ERK-mediated phosphorylation of NPC regulates the nuclear export but not the nuclear import (Fig 1). As the range of EC10 and EC90 becomes narrow, Hill coefficient is increased. Therefore, a reduction in EC90 level caused by ERK-mediated regulation of NPC enabled the dynamics of ERK translocation to be changed from graded to switch-like. Indeed, knockdown of nucleoporin 153, one of the relevant components of NPC that is most effectively phosphorylated by ERK, altered the dose response of nuclear ERK from switch-like to graded [14]. Thus, our model could recapitulate the essential dynamics of the EGF signaling pathways, suggesting that our model can be used for further simulation analysis.

### Effects of intrinsic and extrinsic noise on heterogeneity in nuclear ERK response

**Fig 3:**
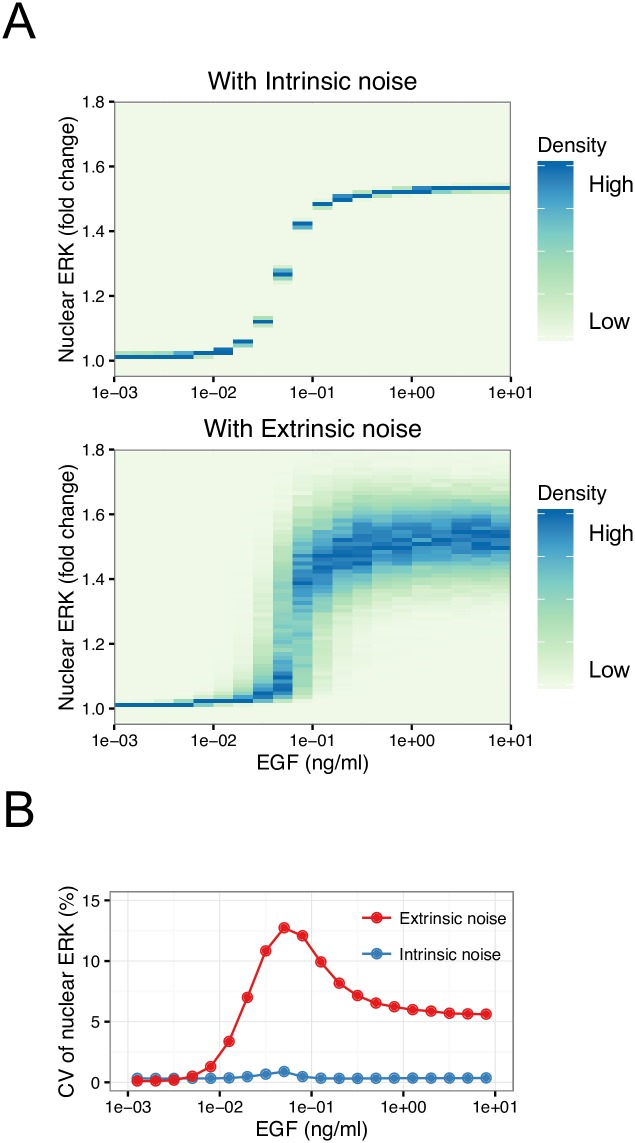
Effects of intrinsic and extrinsic noise on heterogeneity in nuclear ERK. Distributions of fold changes in nuclear ERK with (A) intrinsic or (B) extrinsic noise are shown. (C) CV of nuclear ERK in simulation results with intrinsic and extrinsic noise.

To investigate the effects of intrinsic and extrinsic noise on heterogeneity in nuclear ERK activity, we implemented simulations with either intrinsic or extrinsic noise (see Materials and Methods for details). In this study, the intrinsic and extrinsic noise were defined as fluctuation in reactions and protein variability, respectively. Here, fluctuation in the reactions means that biochemical reaction occurs stochastically, which can be simulated using the Gillespie algorithm [41]. On the other hand, protein variability means that there are differences in the levels of proteins between individual cells, and the noise level is represented by the coefficient of variation (CV). In these simulations, we used a typical CV value of protein variability, 30%, as a representative value of extrinsic noise [8,42]. The distributions of peak levels of nuclear ERK obtained from simulations with intrinsic or extrinsic noise are shown in Fig 3. The distribution with extrinsic noise was clearly broader than that with intrinsic noise at high concentrations of EGF (Fig 3A). For statistical comparison, the CV of nuclear ERK was calculated from simulated distributions. The CV of nuclear ERK with extrinsic noise was higher than that with intrinsic noise (Fig 3B), suggesting that extrinsic but not intrinsic noise contributed to the heterogeneity in nuclear ERK activity.

### Estimation of protein variability by direct comparison of experiments with simulations

**Fig 4:**
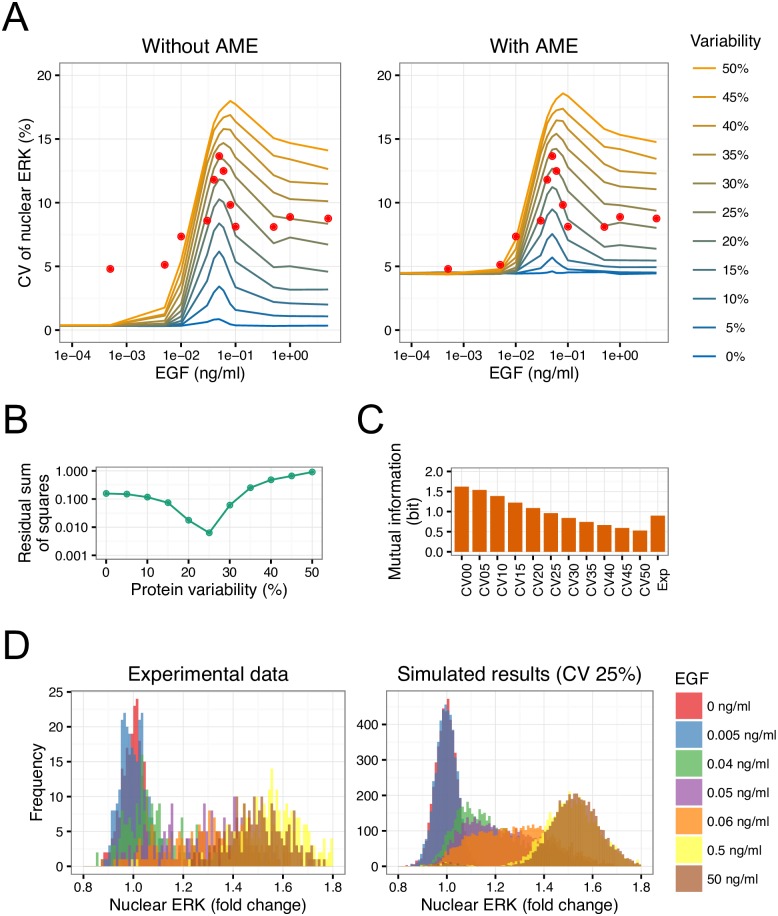
Direct comparison of simulation results with experimental data. (A) CV of simulated nuclear ERK without/with AME are shown. Points represent experimental data [14]. (B) The residual sum of squares between simulation results with AME and experimental data. (C) Mutual information between EGF concentration and nuclear ERK was calculated from simulation results and experimental data [14]. (D) Distributions of nuclear ERK in simulation results at 25% CV of protein variability and experimental data [14].

The variability of proteins between individual cells can be measured by various experimental methods. However, it is still difficult to measure variability of all protein species present in a mammalian cell. To estimate all protein variability in the EGF signaling pathway, we directly compared simulations with experiments. In addition to biological noise, the observed data included several measurement errors derived from measurement principles and setups. Here, such measurement errors were defined as the Apparent Measurement Error (AME), which was determined by our newly developed method (details are described in Supporting Information). As shown in S3 Fig, by applying AME, the distribution of nuclear ERK in simulations corresponded to the observed data [14]. Using the identified AME, we estimated the variability of all proteins in the EGF signaling pathway. First, simulations with both types of noise were implemented under different concentrations of EGF when the CV of protein variability changed from 0% to 50%. The resulting distributions of fold changes in nuclear ERK without and with AME are shown in S4 and S5 Figs, respectively. The CV of nuclear ERK response was calculated for statistical comparison of these simulation results with experimental data [14]. As shown in Fig 4A, AME strongly affected the distributions at low EGF concentration (< 0.01 ng/mL) but had little effect at high concentrations, and by applying AME the CV of nuclear ERK corresponded to the pattern of experimental data. Simulation results at 25% CV of protein variability showed excellent agreement with experimental data (Fig 4A and 4B). For further quantitative comparison, we calculated mutual information, which has been proposed as a good metric to characterize fidelity for a biological system [43]. As shown in Fig 4C, the mutual information between EGF and nuclear ERK also showed that 25% CV of protein variability was the best fit to the experimental value [14]. The distributions of nuclear ERK in simulation results at 25% CV of protein variability were also consistent with experimental data (Fig 4D). Thus, our new method using AME made it possible to predict variability of signaling proteins from only signal output data.

### Effects of covariation among proteins under initial conditions on heterogeneity in ERK

**Fig 5:**
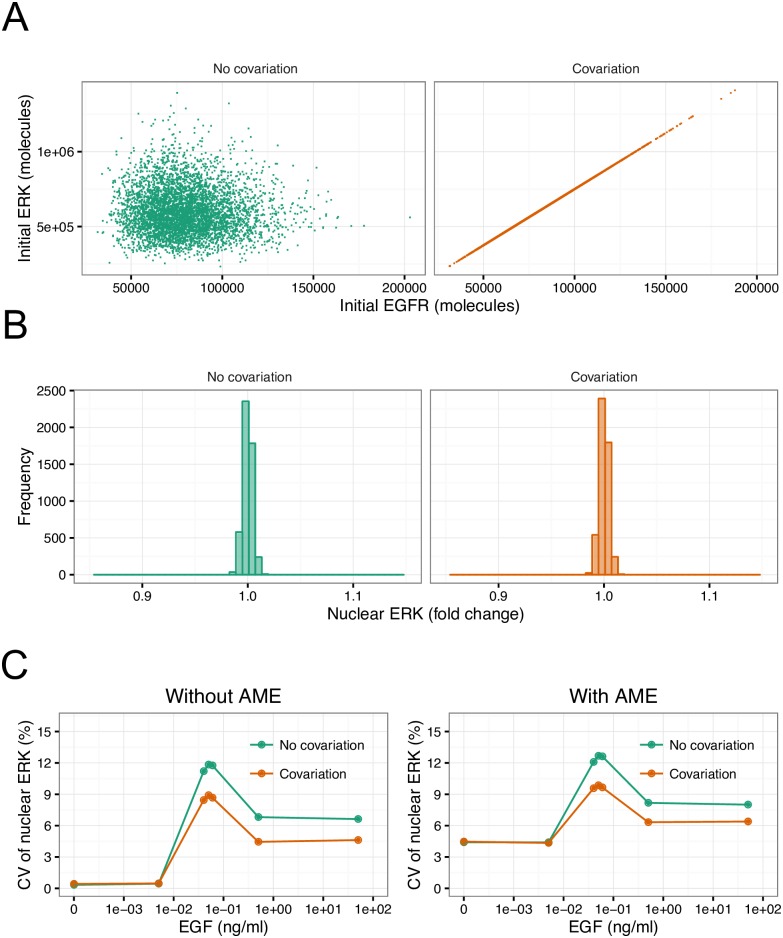
Effects of covariation among initial proteins on heterogeneity in ERK. (A) An example of the relationship among proteins under initial conditions with and without covariation. Points represent different simulation data. (B) The distributions of steady-state levels of nuclear ERK at 25% CV of initial proteins under conditions of no stimulation with and without covariation. (C) CV of peak levels of nuclear ERK with and without covariation with or without application of AME.

In our simulations, extrinsic noise was reproduced by sampling initial proteins randomly from a log-normal distribution. However, it has been reported that individual cells have different expression capacities, leading to variations in the levels of proteins in a correlated manner [6]. To investigate the effects of such covariation among proteins on heterogeneity, we implemented simulations in which all protein levels under the initial conditions were correlated (Fig 5A). The covariation among proteins did not influence the distribution of nuclear ERK at the steady-state level without EGF stimulation, as shown in Fig 5B. However, at high concentrations of EGF, CV of nuclear ERK with covariation was lower than that with covariation (Fig 5C). This tendency for covariation to suppress the heterogeneity in nuclear ERK was found regardless of the application of AME (Fig 5C). Thus, our simulation results suggested that covariation among proteins is involved in heterogeneous cellular responses in the EGF signaling pathway.

### Regulation of heterogeneity in nuclear ERK due to protein variability

**Fig 6:**
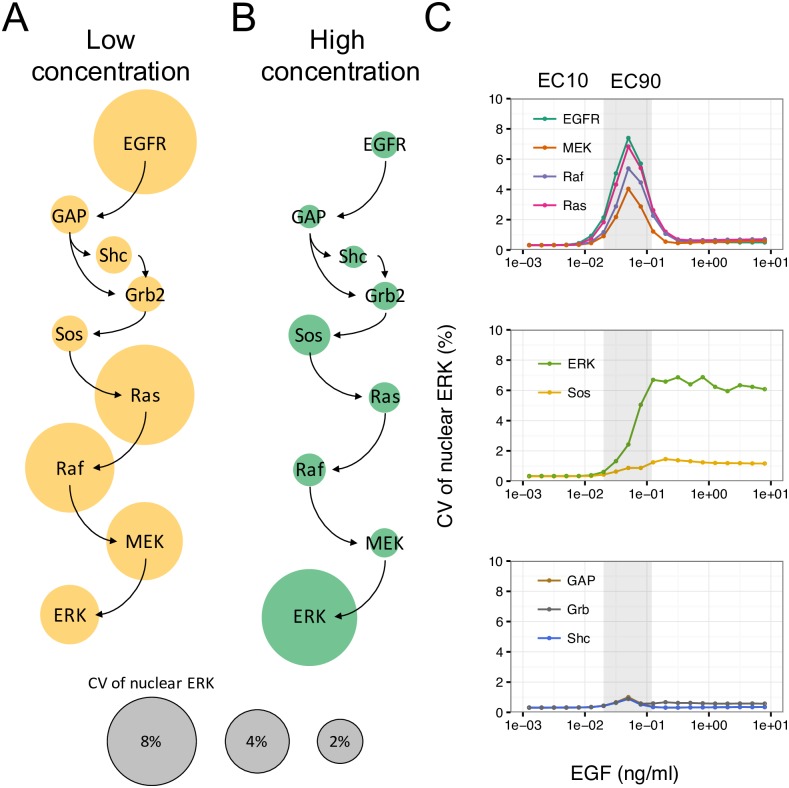
Effects of protein variability of each species on heterogeneity in nuclear ERK. CV of nuclear ERK with changes in protein variability of each species at (A) low (0.05 ng/mL) and (B) high (50 ng/mL) concentrations of EGF. Circle size represents CV of nuclear ERK. (C) CV of nuclear ERK at various concentrations of EGF when changing protein variability of each species. The patterns of CV were classified into three types. CV of 25% was used as protein variability of each species. The gray region represents the area from EC10 (0.02 ng/mL) to EC90 (0.12 ng/mL) calculated from Fig 1D.

To investigate the contribution of each molecular species to heterogeneity in nuclear ERK, we implemented simulations by changing the variability of each protein. The effects of the variability of each protein on heterogeneity in nuclear ERK at low (0.05 ng/mL) and high (50 ng/mL) concentrations of EGF are shown in Fig 6A and 6B, respectively. At lower EGF, variability of EGFR, Ras, Raf, and MEK generated marked heterogeneity in nuclear ERK, whereas variability of ERK and Sos generated large degrees of heterogeneity at higher EGF concentrations. These results suggest that different proteins contributed to cellular heterogeneity in nuclear ERK at different EGF concentrations. Therefore, we investigated the contributions of the proteins in the presence of various concentrations of EGF (Fig 6C). The contributions to heterogeneity in nuclear ERK were divided into the following three types: (1) EGFR, Ras, Raf, and MEK; (2) ERK and Sos; (3) GAP, Grb2, and Shc. Species in the first type evoked large heterogeneity between effective concentration (EC) 10 and EC90 (Fig 6C, top), where cells that did and did not respond to EGF stimulus were mixed (Fig 4D). Accordingly, heterogeneity generated by variability of EGFR, Ras, Raf, and MEK would be closely related to the response to EGF stimulus. On the other hand, species in the second type stably generated large degrees of heterogeneity over EC90 (Fig 6C, middle). In this region, heterogeneous responses occurred at high levels of nuclear ERK, indicating that all cells would respond to the stimulus (Fig 4D). Therefore, variability of ERK and Sos had little effect on the response to EGF. Species in the third type showed little heterogeneity at any concentration of EGF (Fig 6C, bottom), and therefore these species had no contribution to the response to EGF in addition to those in type two. This result indicated that only particular species involved in the EGF signaling pathway, i.e., EGFR, Ras, Raf, and MEK, regulate heterogeneity of nuclear ERK in relation to EGF signaling response, i.e., these proteins function as sensitive nodes in the signaling response. In the apoptotic pathway, whether apoptosis was induced or not was not correlated with variability of any single species included in the pathway [8], suggesting that heterogeneity was regulated by variability of at least more than two species. Our simulation results indicated the possibility of predicting EGF signaling response at the single-cell level by knowing the initial concentrations or variability of four species, i.e., EGFR, Ras, Raf, and MEK.

## Discussion

In this study, we developed a novel mathematical model of the EGF signaling pathway integrated with the mechanisms regulating the nuclear translocation of ERK. Although the nuclear translocation of ERK is critical for cell fate decision [44], the dynamics and the regulation mechanism have not been taken into consideration in conventional mathematical models [19–21]. Our model included ERK-mediated regulation of NPC explicitly, which could realize the observed dynamics of nuclear ERK, i.e., switch-like behavior. Our model assumed that nuclear ppERK phosphorylates the NPC in a two-step process, and then ppNPC but not NPC and pNPC mediated the translocation of nuclear phosphorylated ERK to the cytoplasm. The nuclear ppERK positively regulates its own nuclear export through NPC, and therefore activated ERK inhibits its own accumulation in the nucleus, which generated a negative autoregulation of nuclear ERK. Without this negative autoregulation, as phosphorylated ERK was simply distributed in both the cytoplasm and the nucleus through NPC, nERK and pERK showed the same dynamics, i.e., graded response. Thus, ERK-mediated regulation of NPC was responsible for the switch-like response, which may play a crucial role in cell fate decision. Although the details of the molecular basis underlying ERK nuclear translocation are still controversial and our model includes several assumptions regarding the regulatory mechanism between ERK and NPC, we emphasize that our model captures the essential behaviors of ERK in response to EGF stimulation, including time course and dose response.

Next, we investigated the effects of intrinsic and extrinsic noise, i.e., fluctuations in reactions and protein variability, on heterogeneity in nuclear ERK and found that extrinsic rather than intrinsic noise contributed to cellular heterogeneity. Our model assumed the EGF signaling pathway in a mammalian cell in which the volume is typically > 10^−12^ L, which is much larger than yeast or bacteria (10^−16^–10^−14^ L) [45,46]. Therefore, a mammalian cell has a huge number of molecules even if the same concentrations of proteins are present in yeast and bacterial cells. For example, the EGF signaling pathway consists of 10^3^–10^7^ molecules [40] and the HGF signaling pathway consists of 10^4^–10^7^ molecules [9]. On the other hand, *E*. *coli* and yeast cells possess 10^−1^–10^3^ and 10^2^–10^6^ protein molecules, respectively [47,48]. In general, fluctuations due to intrinsic noise are strongly dependent on the number of molecules, i.e., fluctuations are larger with smaller numbers of molecules. Therefore, intrinsic fluctuation is considered to be negligibly small, and extrinsic noise evokes larger fluctuations in our model. In addition, it was reported that the abundance of transcripts in mammalian cells was regulated by extrinsic noise, i.e., cellular state, population context, and microenvironment [49], indicating that extrinsic noise plays a key role in the transcriptional program. Our simulations demonstrated the importance of extrinsic noise in the signaling response. These results suggest that extrinsic noise plays significant roles in mammalian cellular responses.

Using a newly developed method to estimate AME, we could predict that CV of protein variability in the EGF signaling pathway was 25%. In other signaling pathways, ranges of measured CV of signaling proteins were 8%– 75% in the hepatocyte growth factor (HGF) signaling pathway [9], 21%– 28% in the apoptotic intrinsic pathway [8], and 15%– 30% in normally cycling human cells [42]. Thus, estimated CV was included in the range of observed protein variability, indicating that our method is useful for estimating or predicting the variability of all signaling proteins. The AME estimated by our method agreed completely with observed heterogeneity in nuclear ERK without EGF stimulation, suggesting that at the basal level, variability arising from cellular noise was too small to contribute to cellular heterogeneity. Moreover, in our simulations, covariation among proteins under initial conditions suppressed variation in nuclear ERK at high concentrations of EGF. This suggests that heterogeneous signaling responses can be regulated by such covariation in the cell. Further analysis using estimated protein variability showed that distinct species in EGF signaling pathway have different effects on heterogeneity in nuclear ERK, i.e., EGFR, Ras, Raf, and MEK generated heterogeneity related to the signaling response. These particular proteins function as sensitive nodes causing heterogeneous cell responses in the EGF signaling pathway. On the other hand, proteins other than sensitive nodes, i.e., GAP, Grb2, and Shc, could not influence the heterogeneity at any concentrations of EGF. Such differential contribution to heterogeneity may be due to the mechanism of reactions involving each protein in the pathway. In our model, sensitive nodes are involved in enzymatic reactions such as phosphorylation, while insensitive nodes are related to binding–unbinding reaction. This suggests that heterogeneity in signaling responses may be regulated by the type of network edges, i.e., reaction mode in the signaling pathway. In terms of cellular functions, the expression level or the activity of sensitive nodes would be tightly regulated in the cell for appropriate responses in the signaling pathway, as they are strongly involved in the cellular heterogeneous responses. In fact, mutations of EGFR, Ras, and Raf are related to epithelial mesenchymal transition, migration, and tumor invasion of breast cancers, and furthermore MEK mutation was observed in malignant melanoma [50–52]. Thus, sensitive nodes seem to be committed to maintain normal cellular homeostasis. As the expression levels of these sensitive nodes are closely related to the signaling response, knowing these concentrations may make it possible to predict the signaling response at the single-cell level before stimulation.

## Materials and Methods

### Simulation method

Deterministic and stochastic simulations were implemented in this study. Two types of cellular noise, i.e., intrinsic and extrinsic noise, were simulated. The intrinsic noise was defined by fluctuations in reactions, which were realized by a stochastic simulation method (Gillespie algorithm) [41,53]. The extrinsic noise was defined by protein variability between individual cells, which was represented by independently sampling initial values of each protein from log-normal distributed random variables at various CV [8,9,18]. We implemented four types of simulation: 1) without either type of noise; 2) with only intrinsic noise; 3) with only extrinsic noise; 4) with both types of noise. All kinetic parameters were constant among all simulations except the CV of protein variability (S1, S2 and S3 Tables). In the case of stochastic simulations, we performed 5000 simulations per condition to obtain statistically stable results.

### Parameter optimization

The initial conditions of five species, EGFR, Ras, Raf, MEK and ERK, were estimated from experimental data [22,40]. Similarly, six kinetic parameters related to Raf activation were experimentally determined values (S2 Table). Other initial conditions and kinetic parameters were determined by manual parameter tuning as follows. First, kinetic parameters in published models were used as the initial estimates [20,40]. Then, we performed deterministic simulations changing each parameter one by one, and compared the simulation results with experimental data, i.e., time course and dose response of both phosphorylated and nuclear ERK [14]. Repeating this process, we finally determined the parameter set that reproduced the experimental data.

### Mutual information

The mutual information between EGF stimulus and nuclear ERK response was calculated from simulation results and experimental data. Here, the concentration of EGF and nuclear ERK level were used as input signal (S) and output response (R), respectively. Mutual information, I(R; S), was defined by the following equation:

$$I\left(R;S\right)=\Sigma_{s}\Sigma_{R}P\left(R,S\right){\text{log}}_{2}\left(\frac{P\left(R,S\right)}{P\left(R\right)P\left(S\right)}\right)$$ (1)

where *P(R*,*S)*, *P(R)*, and *P(S)* represent the joint probability distribution functions of *S* and *R*, and the marginal probability distribution functions of *S* and *R*, respectively. As the distributions of simulated *S* and *R* were discretized, direct estimates of mutual information using (Eq 1) were biased. To obtain the unbiased solution, we calculated the mutual information by optimizing the discretized size and jackknife sampling, as described previously [54].

### Experimental data

The experimental data used in this research were originally reported in our previous paper [14].

## Supporting Information

S1 Text How to estimate Apparent Measurement Error (AME). (DOCX)

S1 Fig Detailed reaction networks of EGF signaling pathway model. (A) Detailed scheme of EGFR activation. (B) Reaction scheme of Ras/Raf/MEK/ERK pathway. (C) Detailed diagram of autoregulatory control of nuclear ERK translocation. Red numbers represent the reaction numbers shown in S2 Table.
(TIF)

S2 Fig Effect of ERK-mediated regulation of NPC on the dynamics of nucler ERK. EGF dose response of nuclear ERK at fold change level without or with ERK-mediated regulation of NPC. Color bar represents the range of EC10:EC90.
(EPS)

S3 Fig Estimation of AME. (A) Distributions of simulated nuclear ERK with intrinsic and extrinsic noise without EGF stimulus. The line represents the curve fitting result, which is a Gaussian distribution with a mean of 1.0 and CV of 0.35%. (B) Distribution of nuclear ERK without EGF in the observed data [14]. A Gaussian distribution with a mean of 1.0 and CV of 4.4% was obtained by curve fitting. (C) The distribution of simulated nuclear ERK was calculated by applying AME to the simulation results. The curve fitting results show a Gaussian distribution with a mean of 1.0 and CV of 4.4%.
(EPS)

S4 Fig Distributions of fold change of nuclear ERK without AME at different protein variability. CV of protein variability was changed from 0% to 50%. Colors represent different concentrations of EGF.
(TIF)

S5 Fig Distributions of fold change of nuclear ERK with AME at different protein variability. CV of protein variability was changed from 0% to 50%. Colors represent different concentrations of EGF.
(TIF)

S6 Fig Distribution of fold change in nuclear ERK with intrinsic and extrinsic noise at different protein variability without EGF stimulus. CV of protein variability was changed from 0% to 50%.
(TIF)

S1 Table Initial conditions for our model. (XLSX)

S2 Table Biochemical reactions for our model. (XLSX)

S3 Table Rate equations for our model. (XLSX)

S4 Table Nuclear import/export rates of ERK. (XLSX)

S5 Table Fitted values for the distribution of nuclear ERK at different protein variability without EGF stimulus. (XLSX)
