# Supplementary material for: Modeling Cellular Noise Underlying Heterogeneous Cell Responses in the Epidermal Growth Factor Signaling Pathway
Source: PLoS Comput Biol. 2016 Nov 30;12(11):e1005222. doi: 10.1371/journal.pcbi.1005222 (PMC5130170; doi:10.1371/journal.pcbi.1005222)
Supplement: S1 Text — (DOCX) [file pcbi.1005222.s001.docx]

**Supporting Information**

**How to Estimate Apparent Measurement Error (AME)**

To compare simulations and experiments directly, it was necessary to fill the gap between both data sets. Previously, Elowitz et al. proposed that both intrinsic and extrinsic noise make contributions to the total noise [1]. The intrinsic and extrinsic noise represent fluctuations in the reactions and variability of protein levels between individual cells, respectively. The experimental data had several measurement errors derived from the variously stochastic nature arising from measurement principles and measurement setups. However, it is difficult to distinguish and identify the measurement errors arising from each element [2]. Thus, in this study, these effects were integrated and simply defined as Apparent Measurement Error (AME). We considered that variation in experimental data included three types of noise, i.e., intrinsic noise, extrinsic noise, and AME, and the total variation was represented by the following equation,

${CV}_{observed}^{2}={CV}_{intrinsic}^{2}+{CV}_{extrinsic}^{2}+{CV}_{AME}^{2}$ $\mathrm{CV}_{\mathrm{observed}}^{2}=\mathrm{CV}_{\mathrm{intrinsic}}^{2}+\mathrm{CV}_{\mathrm{extrinsic}}^{2}+\mathrm{CV}_{\mathrm{AME}}^{2}\mathrm{eq}$ $\mathrm{CV}_{\mathrm{observed}}^{2}=\mathrm{CV}_{\mathrm{intrinsic}}^{2}+\mathrm{CV}_{\mathrm{extrinsic}}^{2}+\mathrm{CV}_{\mathrm{AME}}^{2}\mathrm{eq}$ (1)

where, CV and subscript represent the coefficient of variation and origins of noise, respectively. We estimated a function to fill the gap between simulations and experiments based on Eq (1).

Under our experimental conditions, nuclear ERK at the single-cell level without EGF stimulus was sustained over a short time (~ 30 min), regardless of the expression level of ERK [3]. Our simulations without EGF also indicated a steady-state level of nuclear ERK even if the expression level of ERK varied between individual cells (Figures S3A and S6). Therefore, we considered that the variation arising from extrinsic noise was negligible in our experimental condition. The total variation of nuclear ERK without EGF, except for extrinsic noise, was eventually represented as follows.

${CV}_{observed}^{2}={CV}_{intrinsic}^{2}+{CV}_{AME}^{2}$ (2)

Using Eq (2), we estimate the AME function based on our experiments and simulations. Simulated distributions of fold change in nuclear ERK without EGF stimulation, regardless to protein variability, showed Gaussian distributions with a mean of 1.0 and CV of about 0.35% (Figure S3 and Table S5). On the other hand, the distribution of fold change of nuclear ERK without EGF stimulation calculated from experimental data [3] showed a Gaussian distribution with a mean of 1.0 and CV of 4.4% (Figure S3B). These results implied that the function of AME has a Gaussian distribution. Next, we calculated CV of AME using those data based on Eq (2). As a result, a Gaussian function with mean of 1.0 and CV of 4.4% was identified as the AME. Applying AME to simulation results, the distribution of simulated nuclear ERK without EGF stimulation was converted to an experiment-like distribution (Figures S3B and S3C). If simulation methods or experimental conditions are different, the type of AME function would be different. Thus, our new developed method could provide the better estimation of AME.

**References**

[1] Elowitz MB, Siggia ED, Levine AJ, Swain PS. Stochastic Gene Expression in a Single Cell. Science 2002;297:1183–7. doi:10.1126/science.1070919.

[2] Watabe M, Arjunan SN, Fukushima S, Iwamoto K, Kozuka J, Matsuoka S et al. A computational framework for bioimaging simulation. PLOS One 2015;10(7), e0130089.

[3] Shindo Y, Iwamoto K, Mouri K, Hibino K, Tomita M, Kosako H, et al. Conversion of graded phosphorylation into switch-like nuclear translocation via autoregulatory mechanisms in ERK signalling. Nat Commun 2016;7:10458. doi:10.1038/ncomms10485.
