# Supplementary figures and images for: Modeling Cellular Noise Underlying Heterogeneous Cell Responses in the Epidermal Growth Factor Signaling Pathway
Source: PLoS Comput Biol. 2016 Nov 30;12(11):e1005222. doi: 10.1371/journal.pcbi.1005222 (PMC5130170; doi:10.1371/journal.pcbi.1005222)

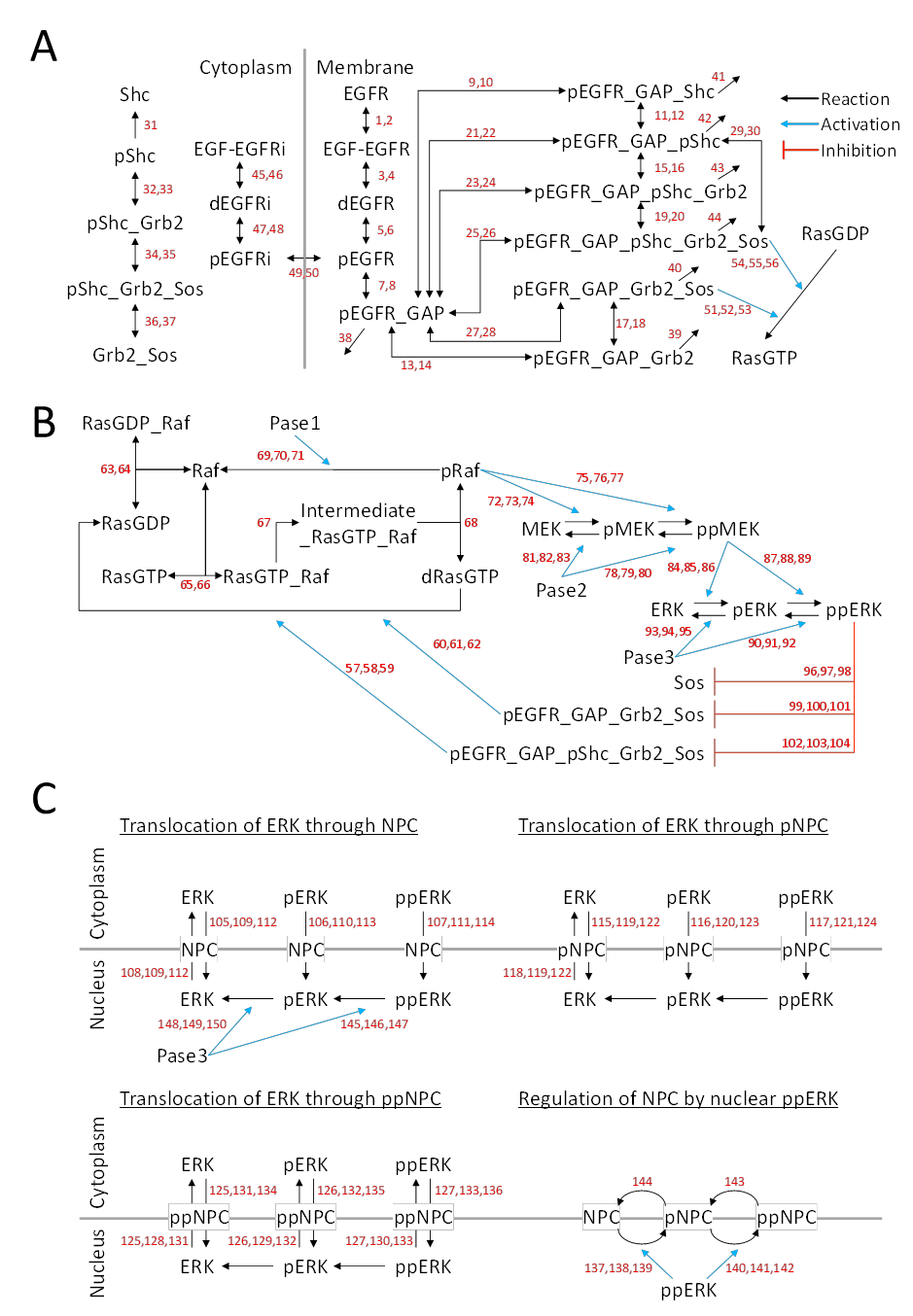

Supplement: S1 Fig — (A) Detailed scheme of EGFR activation. (B) Reaction scheme of Ras/Raf/MEK/ERK pathway. (C) Detailed diagram of autoregulatory control of nuclear ERK translocation. Red numbers represent the reaction numbers shown in S2 Table. (TIF) [file pcbi.1005222.s002.tif]

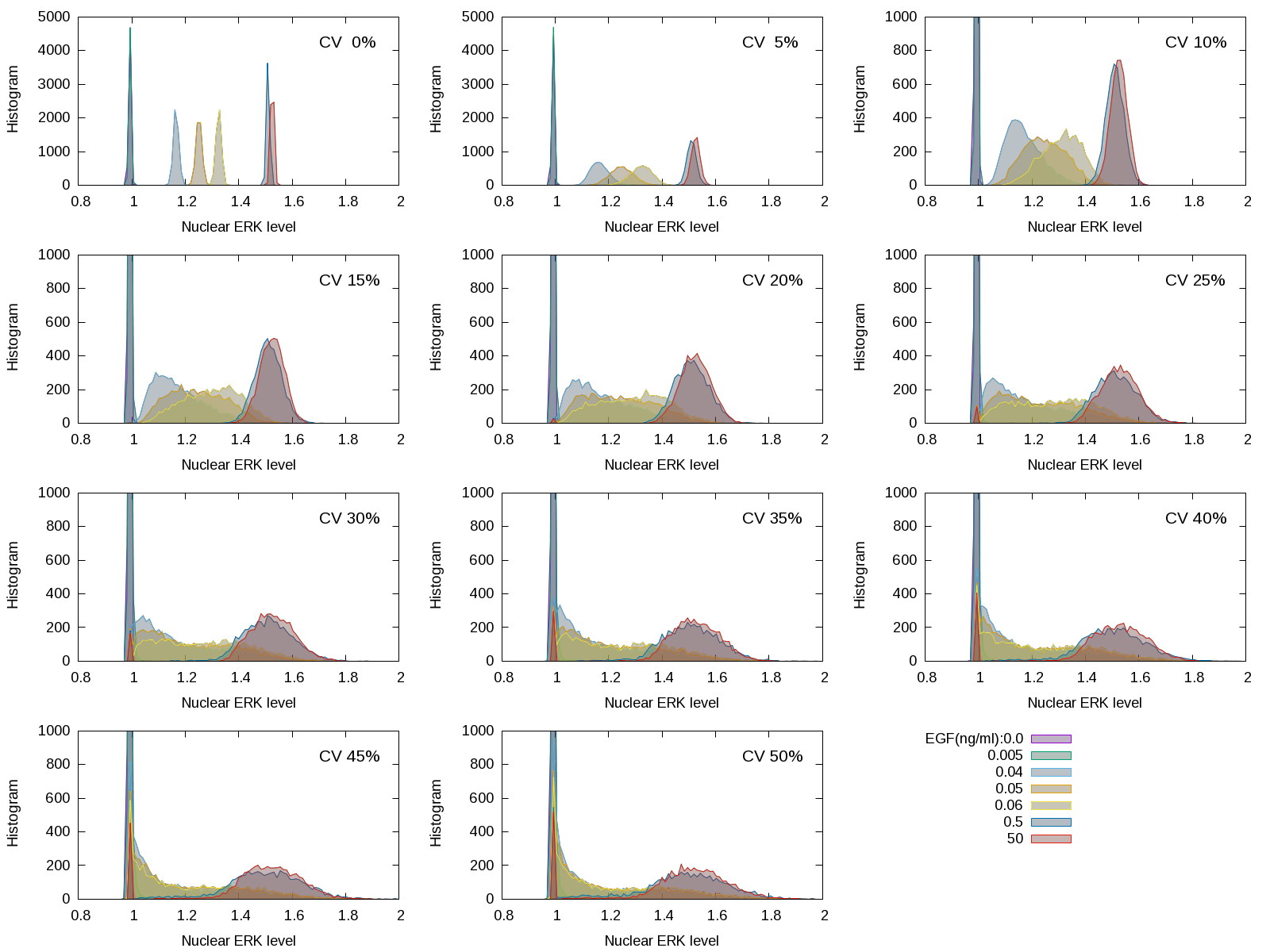

Supplement: S4 Fig — CV of protein variability was changed from 0% to 50%. Colors represent different concentrations of EGF. (TIF) [file pcbi.1005222.s005.tif]

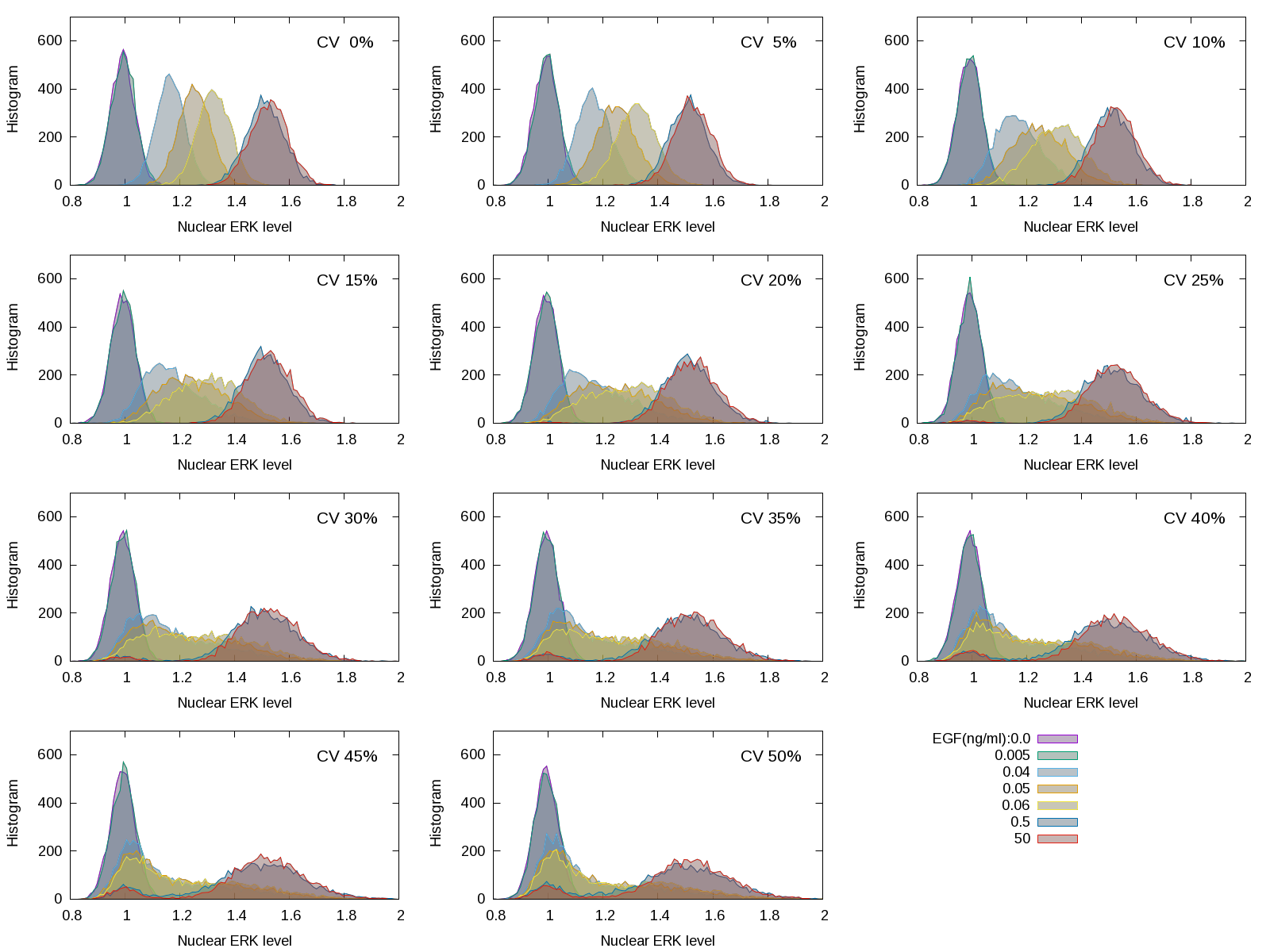

Supplement: S5 Fig — CV of protein variability was changed from 0% to 50%. Colors represent different concentrations of EGF. (TIF) [file pcbi.1005222.s006.tif]

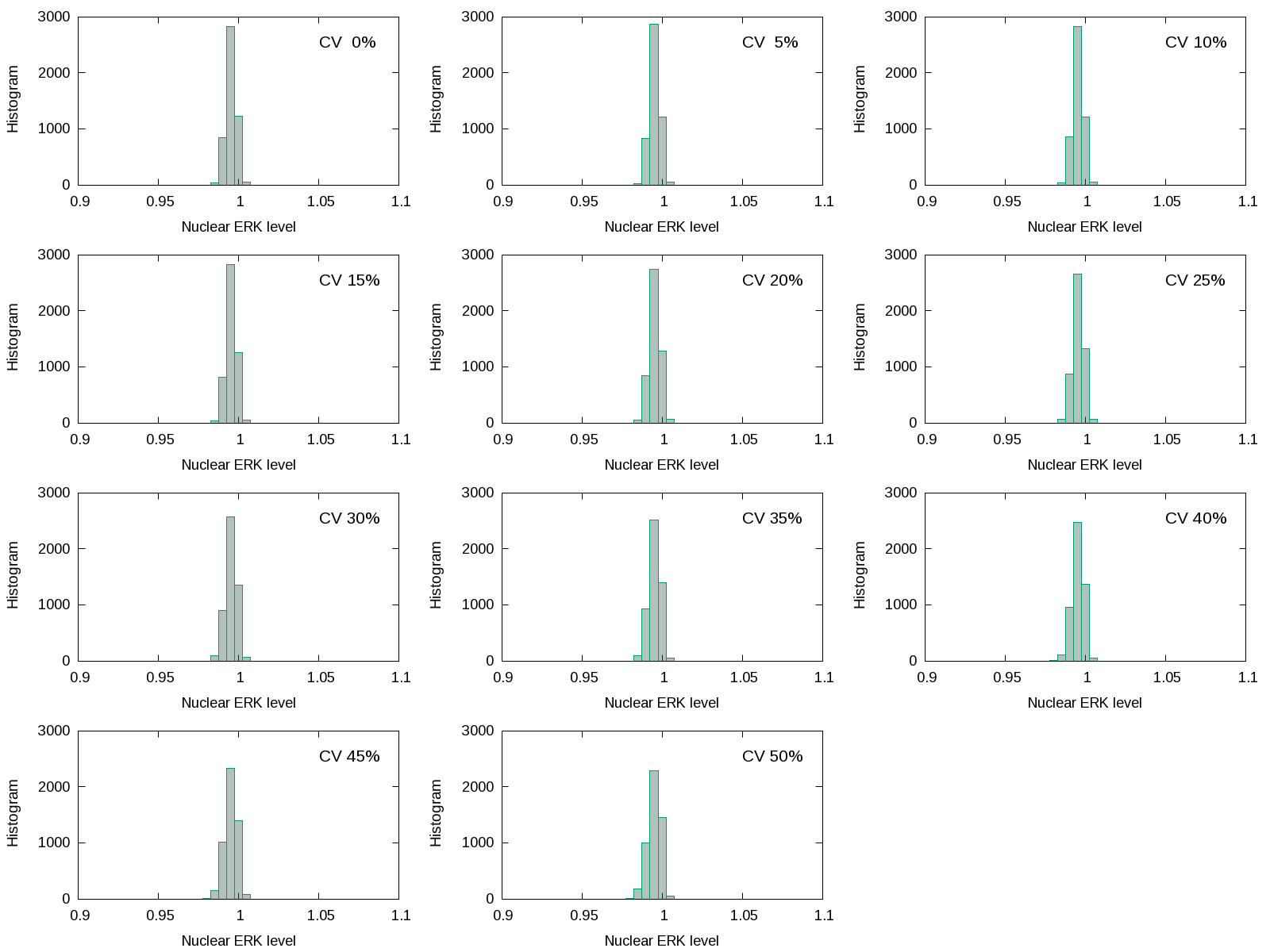

Supplement: S6 Fig — CV of protein variability was changed from 0% to 50%. (TIF) [file pcbi.1005222.s007.tif]
